# Spontaneous Coronary Artery Dissection (SCAD): A Series of 7 Cases, Experience of the University Hospital Center Mohammed VI, Oujda, Morocco

**DOI:** 10.1155/2018/1964394

**Published:** 2018-10-02

**Authors:** I. Benahmed, A. El Kasimi, H. Laachach, N. Ismaili, N. elouafi

**Affiliations:** ^1^Cardiology Department, University Hospital Center Mohammed VI, Oujda, Morocco; ^2^Laboratory of Epidemiology, Clinical Research and Public Health, Faculty of Medicine, University of Mohammed First, Oujda, Morocco

## Abstract

Spontaneous coronary artery dissection is a less known pathology by the cardiologists and represents a real challenge especially to the interventional cardiologist. The positive diagnosis is based on the visualization of intimal flap with the false lumen by intracoronary imaging means. This entity particularly interests young people without classic cardiovascular risk factors of atherosclerosis and female during the peripartum period. We report, in this paper, our experience in the University Hospital Center of Mohammed VI, Oujda, Morocco, about 7 cases diagnosed by coronary angiography during 3 years of practice while comparing our results with data from large series published in the literature. The purpose of this work is to draw more attention to this particular pathology that is becoming more and more common.

## 1. Introduction

Spontaneous coronary dissection is defined as a nontraumatic, noniatrogenic separation of the walls of the coronary artery [1]. The clinical presentation is variable but dominated by the ACS. The diagnosis is based on viewing the false lumen by intracoronary imaging means. We report the experience of the catheterization laboratory at our center which helped to highlight seven cases of spontaneous coronary dissection.

## 2. Materials and Methods

A retrospective database was analyzed from Mohammed VI University Hospital Center Cath lab. Among 2000 cases of coronary angiography performed over a period of 3 years, 7 cases of spontaneous coronary dissection were diagnosed. The follow-up of these cases was assured from the discharge day to December 2017 in order to register any event like angina recurrence, acute coronary syndrome, the need of hospitalization, and death. The clinical and paraclinical data of our patients were grouped in Table 1.

## 3. Results

We indentified 7 patients with SCAD over the past 3 years (from September 2014 to October 2017) diagnosed by coronary angiography in Cath Lab of University Hospital Center of Mohammed VI by visualizing the radiolucent intimal flap. Baseline characteristics of these patients are described in Table 1. There were 6 men and 1 woman; the average age was 58, 85 (range 28–76) years. There were some cardiovascular risk factors in common such as male gender, active smoking, and obesity. Clinical presentation was acute coronary syndrome in 6 cases, and the right coronary artery was involved in 5 cases out of 7.

SCAD diagnosis was made by coronary angiography procedure; we only indentified type 1 by the visualization of a radiolucent intimal flap.

**Figure 1:**
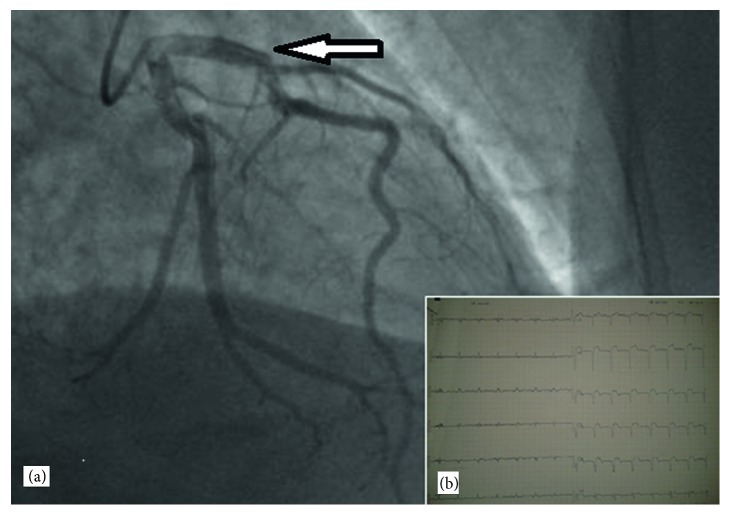
(a) coronary angiography demonstrating a dissection of the left descending artery (white arrow) and (b) electrocardiogram of our patient showing an ST segment elevation anterolateral leads (from V1 to V6, DI and AvL).

**Figure 2:**
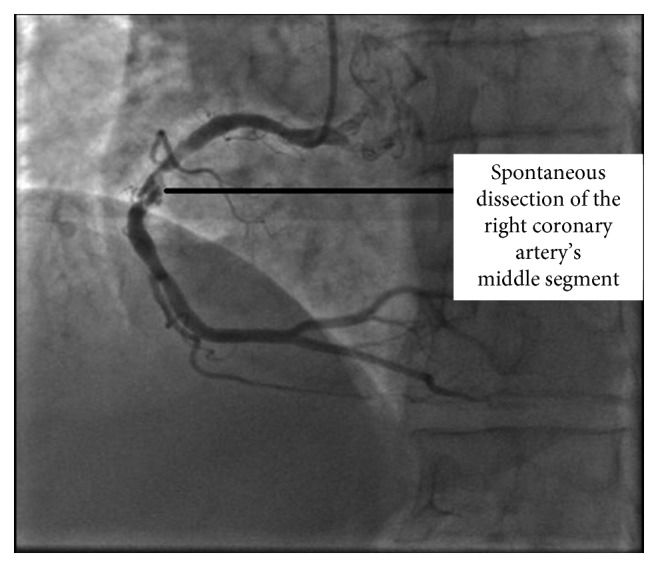
Coronary angiography image of our patient showing a localized spontaneous right coronary artery dissection to its middle segment with atheromatous aspect.

**Figure 3:**
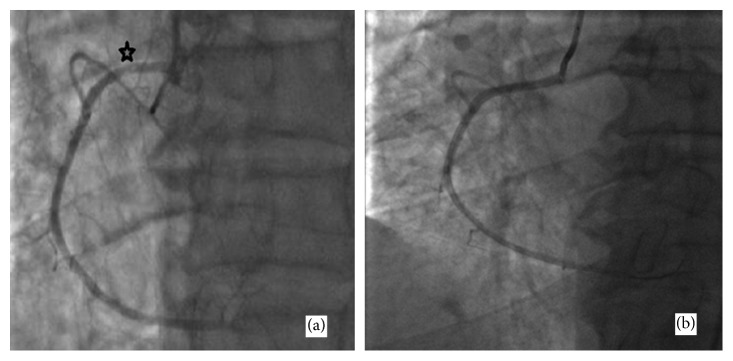
Coronary angiography image of our patient (case 2) showing spontaneous coronary artery dissection of the RCA's proximal segment (a) and the result after PCI with a DES (b).

Two patients received thrombolytic therapy in the acute phase; in the first case, we identified a long dissection of the left anterior descending artery from the ostium to the middle segment with TIMI III flow (Figure 1), and in the sixth case, coronary angiography showed a dissection of the right coronary artery's middle segment (Figure 2). Percutaneous coronary intervention was performed in case number 2 due to ongoing angina pectoris, and we successfully placed a 3/22 mm drug eluting stent in the right coronary artery (Figure 3).

**Figure 4:**
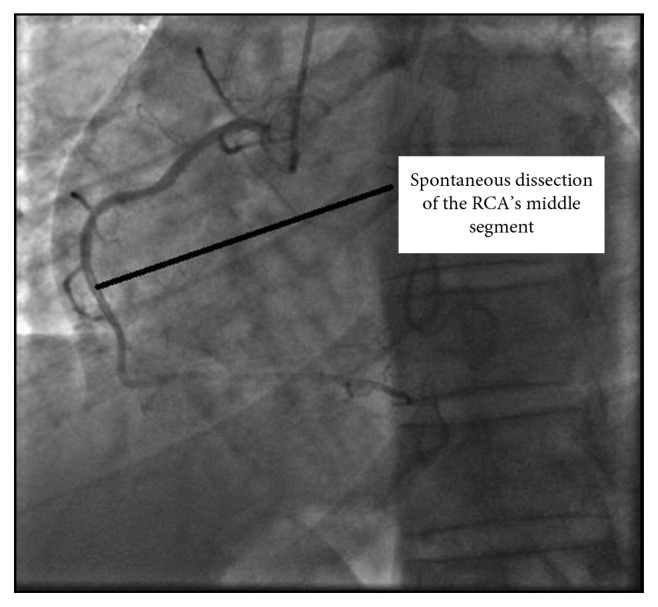
A coronary angiography image showing spontaneous dissection of mid-Right coronary artery.

**Figure 5:**
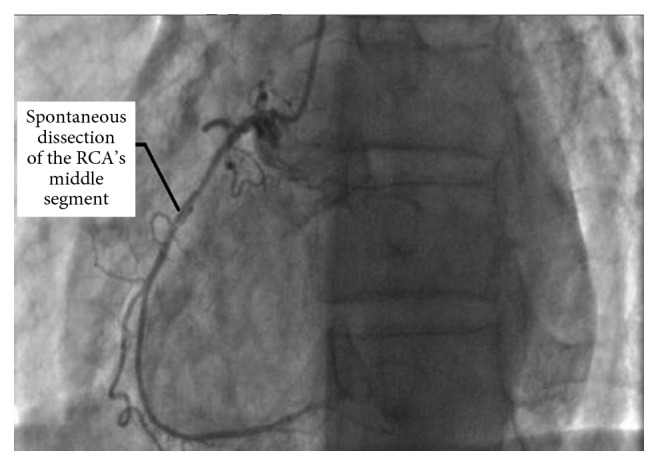
Spontaneous right coronary artery dissection localized to the middle segment.

**Figure 6:**
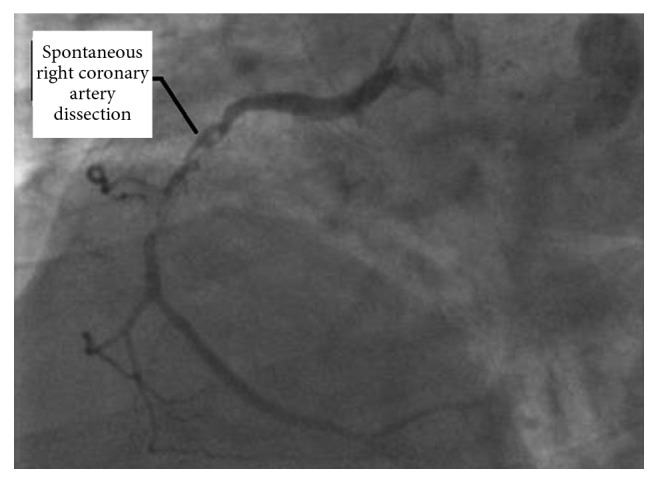
Coronary angiography imaging revealing a long dissection with tight stenosis at mid-right coronary artery with an atheromatous aspect.

**Figure 7:**
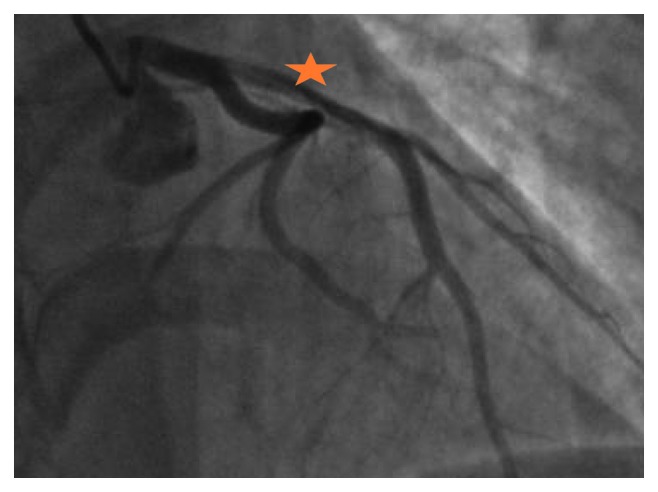
Coronary angiography image showing a spontaneous dissection of the left anterior descending artery of its ostiale and proximal segments.

All our patients received medical treatment base on dual antiplatelet therapy, statin, betablockers, and angiotensin-converting enzyme inhibitor with good outcome (Figures 4–7).

The mean follow-up duration was 16 (2–29) months. No angina recurrence or major cardiac event was registered during the follow-up.

## 4. Discussion

Spontaneous coronary artery dissection (SCAD), also called intramural hematoma or hemorrhage, or dissecting aneurysm, is a very rare pathology responsible for acute coronary syndrome in young and particularly female patients [2]. The incidence of this pathology is 0.1–1.1% of patients referred for coronary angiography [3]. It predominates in young, female patients without risk factors for atheromatous disease and especially in the peripartum period. However, we registered a predominance of male sex in our series with a relatively high average age (58.85) with extremes ranging from 28 to 76 years and a single case of a young man.

This pathology can be responsible for significant morbidities such as myocardial infarction, ventricular rhythm disorders, and sudden death [4, 5]. The first case reported in 1931 was discovered during an autopsy for a sudden death of a 42-year-old patient. [2, 6]. Since this date until 2013, more than 490 cases of DSAC have been reported [7].

The clinical presentation varies from simple angina to sudden cardiac death. It depends on the extent of the dissection, its location, and the degree of myocardial ischemia [8, 9]. The most common clinical presentation is similar to acute coronary syndrome [10]. In our series, we recorded a case of stable angina and 6 cases of acute coronary syndrome.

The diagnosis is made by coronary angiography viewing a type 1 SCAD: a typical aspect of a double lumen with an intimal radiolucent flap [11].

Saw proposed a simple classification system for SCAD based on angiographic analysis [12]. Type 1 describes the pathognomonic multiple radiolucent lumen with contrast wall staining. In the most common variant, type 2 SCAD, a long diffuse (typically > 20 mm) smooth stenosis is noted with abrupt change in the caliber of the involved segment. There is smooth tapering followed by reverse tapering more distally. Type 3 describes focal or tubular stenosis that mimics atherosclerotic plaque. Intracoronary imaging is needed in such cases to confirm SCAD [12, 13].

Other modern techniques may be useful in case of doubt, such as intravascular ultrasound, optical coherence tomography, or coronary CT angiography. The most involved artery is LAD in almost 75% of the cases described, followed by right coronary artery (RCA) in 20% of the cases, and the left main coronary artery in 6 to 12% of cases whereas the circumflex artery participates in it less frequently [14, 15]. However, our series shows a predominance of damage to the right coronary artery in 5 out of 7 cases.

On the etiological level, patients with spontaneous coronary dissection can be grouped into two subgroups: atherosclerotic group and nonatherosclerotic group that include diseases and factors predisposing to arterial injury (connective tissue disorders, drug intake, hypertension, cocaine abuse, vigorous exercise, etc.) [16]. However, a significant amount of cases remain undiagnosed with no underlying condition and are being classified in an idiopathic group.

Our series presented features in which there was a clear male predominance with advanced age with presence of risk factors for atheromatous disease and a single case of a patient with active rheumatoid arthritis. In other cases, data of coronary angiography were in favor of atheromatous cause in 5 cases due to the presence of a diffuse atherosclerotic aspect of artery walls.

There is no specific guideline on how to manage patients with SCAD. Treatment options for SCAD include medical therapy, percutaneous coronary intervention (PCI), or coronary artery bypass graft surgery (CABG) [17]. Medical treatment should be initiated in patients with spontaneous dissection localized to the middle or distal segment of the coronary arteries with monotruncular involvement and obliteration of less than 50% of the arterial lumen diameter [18–20]. Spontaneous healing under medical treatment alone has been described by several authors [21–27]. The decision of revascularization should be made carefully and selectively: interventional percutaneous treatment is required in case of involvement of the proximal segment of a coronary artery with persistent angina or anginal recurrence; ischemia limited to a small territory and patients who presented at acute phase [28–30]. CABG may be proposed for patients with hemodynamic instability, multivessel involvement, left main coronary artery involvement, and after failure of angioplasty [22, 31–33].

In the absence of a catheterization room and in the event of an emergency, thrombolysis presents the first choice of revascularization of an acute coronary syndrome with ST segment elevation, which can cause a real danger in case of spontaneous dissection of the artery coronary because of the risk of its extension by increasing intramural bleeding [34, 35] and may even be fatal.

In our series, all our patients received medical treatment based on a double antiplatelet aggregation, statin, angiotensin-converting enzyme inhibitor, and betablocker. PCI was used in one case in addition to medical treatment, giving the presence of extensive dissection from the ostium of the right coronary artery with persistent pain.

Thrombolysis was performed in two cases. In the first case, we had an extensive dissection of the left anterior descending artery which was respected due to the presence of a good downstream flow and the absence of angina recurrence. The second patient had a localized dissection at the second segment of the right coronary artery with a good distal flow.

The intrahospital evolution was favorable. During follow-up, we registered no anginal recurrence, major cardiac event, or death case in our series.

The long-term prognosis is generally good at 95% after a follow-up of 2 years [36]. Several authors reported excellent long-term follow-up in patients who were managed conservatively [16, 37–41].

## 5. Conclusion

Spontaneous coronary dissection is a rare entity that is often overlooked by cardiologists and presents a challenge for interventional cardiologists for both diagnosis and treatment. Our series presented some peculiarities: the relatively advanced age of the patients, the clear predominance of the male sex, the atheromatous etiology, and the involvement of the right coronary artery. All patients have evolved under medical treatment; one patient underwent percutaneous coronary intervention due to the persistence of angina and the presence of an occlusion of the ostium of the right coronary artery.

## Figures and Tables

**Table 1:** Summary table of the different clinical, echocardiographic, and coronary angiographic characteristics of our patients.

Case	Age	CVRF	Gender	Clinical presentation	LVEF	Thrombolysis	Involved coronary artery	Treatment	Follow-up (in months)
1	76	Age, gender	M	Anterolateral STEMI	28%	Yes	LAD	Medical	25
2	67	Age, gender, obesity	M	Inferior STEMI	45%	No	RCA	Medical and PCI	29
3	53	Age, gender, obesity, smoking	M	NSTEMI	64%	—	RCA	Medical	18
4	62	Age, gender, heavy smoking, obesity, abdominal obesity	M	NSTEMI	51%	—	RCA	Medical	16
5	66	Age, diabetes, hypertension, obesity, abdominal obesity	F	Angina of effort	62%	—	RCA	Medical	13
6	60	Age, gender, hypertension	M	STEMI	54%	Yes	RCA	Medical	13
7	28	Gender, chewed tobacco	M	STEMI	15%	No	LAD	Medical	1

STEMI: ST-elevation myocardial infarcation; M, Male; F, Female; LAD, left anterior descending arteryl; RCA, right coronary artery; LVEF, left ventricular ejection fraction; CVRF, cardiovascular risk factors.

## Data Availability

All data generated and/or analyzed during the current study are included in this article.
